# Memory underpinnings of future intentions: Would you like to see the sequel?

**DOI:** 10.1371/journal.pone.0176624

**Published:** 2017-04-27

**Authors:** Marta Stragà, Fabio Del Missier, Francesco Marcatto, Donatella Ferrante

**Affiliations:** 1 Department of Philosophy and Cultural Heritage, Ca’ Foscari University of Venice, Venice, Italy; 2 Department of Life Sciences, University of Trieste, Trieste, Italy; Hangzhou Normal University, CHINA

## Abstract

In two studies, we investigated the memory underpinnings of future intentions related to past hedonic experiences. Preceding research did not make clear whether the specific memory processes supporting the expression of intentions about the future involve global judgments of the past experience (general affective evaluations formed on-line) or judgments derived from the episodic recollection of the past. Adapting a correlational paradigm previously employed to study future intentions, and applying it to the experience of watching a movie, we comparatively tested the influence of global retrospective evaluations vs. episodic-derived evaluations on future intentions. In Study 1, in which the intentions involved a future experience that was very similar to an overall past one (e.g., seeing the movie sequel), the findings showed that participants relied only on global judgments to form future intentions. In Study 2, in which the global judgment on the past was less diagnostic because the future intentions referred to specific parts of the past experience (e.g., watching a movie centered on a minor character in the previously seen movie), the results indicated that relevant episodic memories provided an essential contribution to the prediction of future intentions. These findings are in agreement with the predictions of the accessibility-diagnosticity framework and they show that global judgments and episodic memories of a past experience contribute differentially to diverse kinds of future intentions.

## Introduction

Last week you watched a movie on TV and found it nice. Now, talking with a friend, you hear that its sequel will be played in your town in some days. Your friend asks you whether you are interested in watching the sequel. What will drive your future intentions? The studies presented in this paper aim to answer this question, tackling the open issue of the identification of memory bases of future intentions about hedonic experiences, a topic that has strong theoretical and applied relevance. In their seminal work, Wirtz and colleagues [1] showed that the intention to repeat a past experience (i.e., the willingness to repeat a holiday) does not depend on the actual experience but on individuals’ memory of that experience (see also [2]). However, research on future intentions has still not made clear what “memory” means in this context, because it has not specified what kinds of memories are used in formulating intentions about future experiences closely related to past ones. Our studies aim to fill this significant gap.

The background of the work reported in this paper stems from the integration of the accessibility-diagnosticity framework [3] with Hastie and Park’s theoretical distinction between on-line evaluations and memory-based judgments [4], complemented by related studies. Although these two frameworks were not originally applied to future intentions about hedonic experiences, we will show that their integration allows for making specific predictions in the future intention domain, predictions that we tested in two empirical studies on a movie experience.

Hastie and Park [4] drew (and empirically supported) a distinction between on-line evaluations of incoming information, which lead to global judgments that can be retrieved at a later stage, and memory-based judgments, which are expressed retrospectively by relying on purposely-retrieved episodic information. Global judgments are routinely formed (for instance when forming an impression about an experience or a person) and used prospectively by default whenever needed, while memory-based judgments are rather infrequent and usually articulated only when a global judgment is not already available. Betsch and colleagues [5] showed also that evaluative summative judgments may be formed implicitly after encoding of value-charged stimuli, while Hermans and colleagues [6] suggested that semantic memory may have the function of assuring access to global evaluations about the options. To summarize, Hastie and Park’s distinction, and later studies, highlighted two potential routes to future intentions: (a) the retrieval of a summative judgment about the past experience that can be used as a basis to express the future intention (default route); (b) the recollection of episodic memories about the past experience and their evaluation to formulate the future intention. However, this view does not fully make clear what the conditions leading to the activation of one or the other mechanism are.

The accessibility-diagnosticity framework is helpful in this respect [3]. This framework has been applied mainly to choices between different brands [7], word-of-mouth effects (e.g., [8]), effect of advertising on decisions (e.g., [9]), effect of measurement in survey research (e.g., [10]), and interference of post-experience information on overall judgments about an experience (e.g., [11]; for a review, see [12]). In the present paper, we aim to extend it to future intention of hedonic experiences. Following the framework, it can be postulated that a given memory source (e.g., a pre-existing global evaluation or a specific episodic memory) will be used to formulate a judgment if it is more accessible and more diagnostic than alternative sources [3], with accessibility defined as ease of retrieval and diagnosticity as the degree to which the input is perceived to accomplish the evaluative goal at hand. Lynch and colleagues [7] hypothesized that information sources are sampled from memory sequentially according to their accessibility, but used only if perceived as sufficiently diagnostic, and that the search process stops when a cumulative diagnosticity threshold is reached [13].

In line with these views, it can be assumed that, in a common hedonic situation, individuals can retrieve two main sources of information from their memory in order to formulate a future intention starting from a past experience: a global judgment about the past experience and a pool of episodic memories related to that experience. Recollection-based judgments of future intentions will be viable only when episodic information is still accessible and can be retrieved (thus, generally after a few days or a week after the experience; see [14, 15]). When the delay is longer and episodic memories fade away, the global judgment will be preferred.

When the delay between the experience and the expression of intentions is short (i.e. within one week), however, both global judgments and episodic memories should be accessible. In that case, if both sources are also equally diagnostic, using the global judgment for the intentions should be the default, considering that retrieving a global judgment is much faster than retrieving more specific relevant information [16] and that evaluating or aggregating the latter kind of information in a judgment may imply additional costs. Converging support comes by fuzzy trace theorists, who advocated the prevailing use of global gist-based (vs. verbatim) representations in reasoning tasks by relying on functional considerations (see e.g., [17, 18]).

Indeed, according to a rational analysis (e.g., [19–21]), future intentions should be grounded on memory-based judgments only when perceived benefits, in terms of more accurate prediction of future enjoyment and satisfaction, exceed the additional retrieval and evaluation costs, albeit the individuals’ degree of rationality still needing to be demonstrated in this specific context. In line with this consideration, when the delay between the experience and the expression of judgments is short but global judgments are not sufficiently diagnostic (i.e., they do not fit the evaluation requested), individuals should retrieve also relevant episodic memories and use them to formulate future intentions (see also [7]).

### Overview of the studies and hypotheses

In two studies, we tested the previously specified hypotheses about the memory bases of future intentions related to the hedonic experience of watching a movie. The movie scenario has been used frequently in previous research (e.g., [22–24]) because it allows exerting experimental control while retaining the ecological validity of a real hedonic experience.

In particular, we tested our hypotheses under a short time frame (i.e., when both global judgments and episodic memories are still accessible) and varying the diagnosticity of the overall evaluations vs. specific episodic memories for expressing intentions (by changing the target of the future intention). We expected that participants would have preferentially used the global judgment when it was diagnostic for the target of future intentions (H1), but that they would have resorted also to relevant episodic memories of the past experience when the global judgment was made less diagnostic by changing the target of intentions (H2) (e.g., [3, 7]).

We tested H1 in Study 1, in which the intentions involved a future experience very similar to an overall past one (e.g., seeing the movie sequel), and H2 in Study 2, in which the future intentions were related only to specific parts of the past experience (e.g., watching a movie centred on a minor character in the previously seen movie). Future intentions were expressed one week after the viewing session, so that both global evaluations and episodic traces were highly accessible and could be easily retrieved (i.e., a short time frame according to [14]; see also [25]). Indeed, there seems to be good agreement on the fact that individuals switch to global judgments when episodic traces are difficult to retrieve (e.g., when the time lag between the experience and the expression of the intention about the future increases; see also [26]).

In our studies, we adapted a correlational paradigm that has been previously used to identify the relative roles of expectancies, online evaluations, and memory on the intention to repeat a hedonic experience [1]. Specifically, our adaptation of this paradigm allowed disentangling the role of global judgment vs. episodic recollection on future intentions, while controlling for the influence of prior expectancies on the actual experience and on both kinds of retrospective evaluations. Indeed, any proper investigation of the memory bases of future intentions needs to control for the influence of expectancies, because they may strongly influence actual enjoyment (e.g., [1, 24, 27]) and retrospective judgments [1, 24, 26]. Failure to do so can lead to misinterpretation of research findings. Moreover, considering both specific memory processes and the role of expectancies will offer a more complete and insightful account of the intention formation process.

## Study 1

### Method

#### Participants

One hundred and twenty undergraduates took part in the study (84% females, age: M = 21.10, SD = 3.93), none of whom had seen the movie before.

All participants provided their informed oral consent prior to inclusion in the studies, during the participants’ enrollment phase (the experimenter registered the participants who gave their consent). Given the low-risk nature of the studies, written consent was not considered necessary. This procedure and the studies were approved by the Human Research Ethics Committee of the University of Trieste in compliance with APA Ethical Guidelines and the Code of Ethical Principles for Medical Research Involving Human Subjects of the World Medical Association (Declaration of Helsinki).

#### Procedure and materials

Participants were recruited after announcements made during course lessons and via the University online message boards. After expressing their willingness to participate in the study, they were randomly assigned to small groups (that ranged from 15 to 30 participants each), and they were invited in a specific date and time in one of the University buildings. The study was carried out in two collective sessions. In the first session, participants were asked to take place in an experimental facility equipped with loudspeakers and cinema screen. Participants were told that we were interested in studying the reception of movies and that they would watch a movie-excerpt. The video was taken from the movie Caramel [28], a 2007 Lebanese comedy/light drama. The movie focuses on the daily lives of five Lebanese women living in Beirut, each of them having their own issues. The movie has been already used in previous research [29–30], showing that it was moderately pleasant and relatively easy to be processed. Indeed, we were interested in testing a common viewing situation, and we wanted to avoid floor or ceiling effects that might result from very pleasant or very bad movie experiences. Moreover, the movie is not well-known in the country in which the study took place, and this reduces the possibility that participants had seen the movie before.

Participants read a short passage of text describing the movie’s plot and then they expressed their expectancies about the movie on a 6-item questionnaire (after [1, 24, 31]). Judgments about expected interest, pleasantness, general involvement, satisfaction, emotional involvement, and enjoyment were provided by ticking 7-point scales ranging from not at all to very much (Cronbach’s α = .89) and an expectancies score was computed by averaging these ratings. Next, participants watched an excerpt of the movie Caramel [28]. The movie was interrupted after twenty-eight minutes, when a scene ended, so that the interruption was not abrupt. Following previous studies, online experience was measured with two methods: online experience sampling (e.g., [32]) and immediate evaluation after the experience (e.g., [24]). Half of the participants rated how pleasant they found the movie every four minutes on a 7-point rating scale, ranging from not at all to very much (see also [22]), while the other half provided an overall rating only once, just after the end of the movie excerpt (see also [24]). In particular, each randomly-assigned participant received one of two different versions of a booklet. In the first version, the booklet included multiple pages with a single question asking how pleasant they found the movie. Every four minutes, after being prompted by the experimenter, participants had to answer this question by ticking the scale point best reflecting their current evaluation and turn the booklet page. Participants with the alternative version of the booklet were presented with multiple pages presenting a single filler question (“Is there any person on the screen right now?”) and, at the end, they rated how pleasant they found the movie. The evaluation breaks during the movie were chosen so that they were sufficiently long-spaced to avoid interrupting the narrative flow, but at the same time sufficiently frequent to ensure a rather comprehensive sampling of the hedonic experience (moreover the breaks did not interrupt important scenes or dialogues). The evaluation ratings of these two groups were then pooled, given that the overall final rating of the latter group did not differ from the last rating of the repeated rating group, *t*(118) = 1.42, *p* = .16 (additionally, the modelling results presented in the next section do not change if the online score is composed by the average of the repeated ratings, showing that they are robust to variation in the assessment of the online experience). After the completion of the first session, we told participants to come for the second session in the same place after one week. Moreover, participants were asked not to talk about the study with other participants/friends and not to watch the rest of the movie or collect information about it.

During the second session of the study, one week after the first, we collected the two memory-related measures and elicited future intentions. The order of the memory-related measures was counterbalanced, with the future intentions measure interleaved to minimize carry-over effects. In the recollection-based (episodic) measure (see also e.g., [23, 4]), participants had to recall as many scenes as possible in ten minutes and then they had to rate each retrieved scene for its pleasantness, vividness, and emotional involvement on 7-point scales, ranging from not at all to very much. A score for each of these three dimensions was then derived by averaging ratings on the first five scenes recalled (Cronbach’s α = .68, .80, and .78, respectively). In the next section, we will present results based on the episodic-derived score of pleasantness over the first five scenes recalled, given that all participants recalled at least five scenes. However, results were similar also when considering the whole set of retrieved scenes (details are reported in the results section and in S1 Appendix). In the global retrospective evaluation of the movie experience, participants were asked to evaluate the movie by answering the same questionnaire used to assess expectancies (see also [1]), but with the verbs in the past tense (Cronbach’s α = .94) and a global retrospective evaluation score was computed by averaging these ratings.

A 6-item questionnaire with 7-point rating scales was used to elicit future intentions. The items were adapted from items that had been already used to investigate future intentions. Starting from questions on the intention to repeat an experience similar to the past one used in previous studies on future intentions [1, 22, 24], we designed four items for the movie context: the willingness to watch the rest of the movie, its sequel, a movie by the same director, with the same actors (ranging from not at all to very much). Moreover, we included one item about the willingness to pay (WTP) in order to see all the movie (seven options: €0, €1-€2, €2-€3, €3-€4, €4-€5, €5-€6, €7, converted in a value between 1 and 7—we note that the item values are not disjunctive and this may have induced a measurement error, but excluding this item from the intention score did not change the results). WTP questions are commonly used in economic psychology and are thought to reflect attitudes toward target items (e.g., [33]). The final item, asking the willingness to suggest the movie to a friend (ranging from certainly not to certainly yes), was derived from consumer research studies in which recommendation to others is used as an indicator of future intentions (e.g., [34]). The future intentions score was obtained by averaging responses to these six items (Cronbach’s α = .89).

### Results and discussion

Participants recalled a high number of scenes (*M* = 8.30, *SD* = 1.68), which were evaluated as vivid (*M*_*vividness*_ = 5.43, *SD* = 1.07), significantly above the mid-point of the scale, *t*(118) = 14.61, *p* < .001. Additionally, we checked that the retrieved scenes were reported in sufficient detail to be considered as based on actual episodic recollection and they were found to be accurate representations of what happened in the corresponding part of the movie. In particular, we considered as a valid episodic recollection a scene report that was sufficiently detailed to allow a precise identification of a specific scene in the movie, explicitly mentioning specific events, places, actions performed, objects, and so on (cf. [35]). Scenes not meeting these requirements were considered as inaccurately reported. Accuracy of a scene report was further assessed by evaluating the correspondence between the report and the actual scene of the movie. Scenes were evaluated by two independent judges with good inter-rater agreement (Cohen’s kappa = .71, computed on a random selection—10%—of retrieved scenes). Disagreements were reconciled through the raters’ joint discussion. The analysis showed that over the 95% of the retrieved scenes were valid episodic recollections. This shows that episodic information was highly accessible and accurate after one week from the experience. The episodic-derived score was computed on the pleasantness ratings of the first (and presumably more accessible) five scenes because all participants reported at least five scenes. However, given that it is possible to argue that some of the scenes that come to mind are more weighted than the other (e.g., [22]) or that the episodic judgment is more dependent on the most pleasant scene (e.g., [36–37]) we did a sensitivity analysis, testing several models with varying number of scenes in order to show that results do not change. Path analysis results presented in the next section did not change even when using the whole set of retrieved scenes to compute the episodic-derived score (see S1 Appendix).

The order of administration of memory measures did not affect episodic-derived evaluations (4.24 vs. 4.41), *t*(118) = 0.81, *p* = .420, global retrospective evaluations (4.66 vs. 4.41), *t*(118) = 1.29, *p* = .199, or future intentions (4.53 vs. 4.25), *t*(118) = 1.37, *p* = .173, so it was not considered any further.

**Fig 1:**
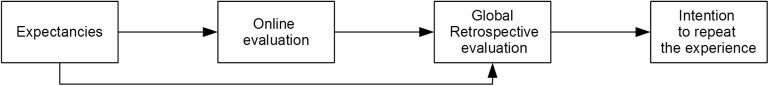
The structure of Wirtz and colleagues model [1].

Following previous studies [1, 2], path analysis was used for hypothesis testing. Models were estimated with the maximum likelihood method of the IBM SPSS AMOS 21 package [38]. We reported indices of fit that are commonly used in literature (the cut-off values that represent a good fit are reported between parentheses; see [39]): χ^2^ (not significant), χ^2^/df (≤ 3), SRMR (≤ .09), RMSEA (≤ .05), CFI (≥ .95). Moreover, we reported three descriptive goodness-of-fit criteria to compare models: the Bayes information criterion (BIC), the Akaike's Information Criterion (AIC) and the Consistent AIC (CAIC). The matrix of correlations between variables is reported in the S1 Appendix. The models were specified by following previous studies [1, 2], which assumed (and observed) a sequential chain of relations between expectancies, on-line evaluation, retrospective evaluation, and intentions (see Fig 1).

**Fig 2:**
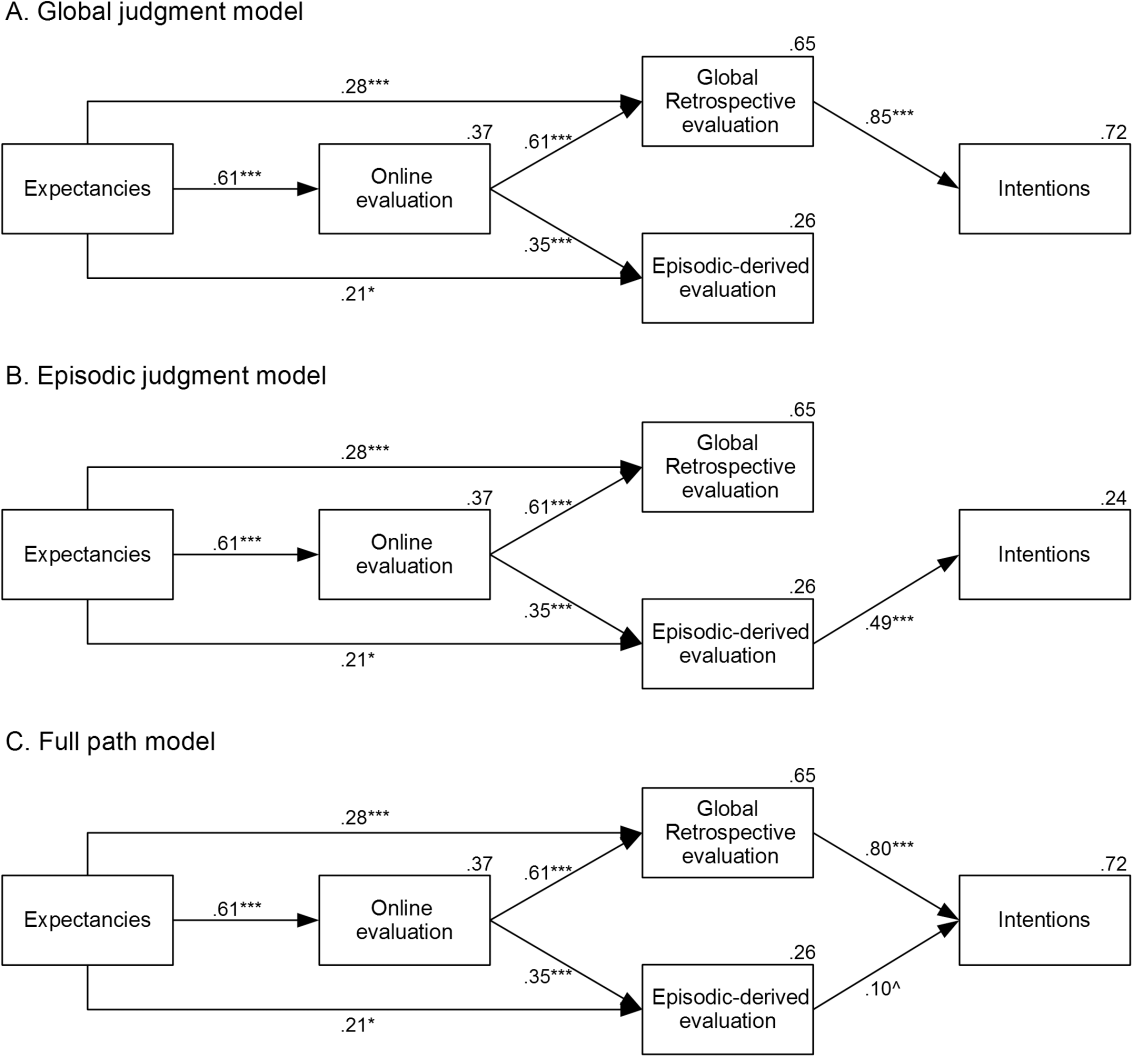
Path analysis models in Study 1. Path analysis models for the test of the global judgment model (panel A) vs. episodic judgment model (panel B) and the full path model (panel C). Numbers close to the arrows are standardized path coefficients. Those above the boxes indicate explained variance (*R*^*2*^). Significance levels are as follows: ^ *p* < .10, * *p* < .05, ** *p* < .01, *** *p* < .001.

However, in order to test the role of global retrospective evaluation and episodic-derived evaluation on the intentions related to similar future experiences, we included in the models the two distinct measures of retrospective evaluation (i.e., global retrospective evaluation and episodic-derived evaluation) and we tested separately two alternative models including a selective link between one of these measures and future intentions. Intentions were supposed to be selectively predicted by global retrospective evaluation in the first model, and by episodic-derived evaluation in the second model. Given that some studies reported that episodic recollection may be used as a basis for the retrospective evaluation formation [14], we initially included a correlational link between the two memory measures. In both the models, this relationship was not significant (β = .16, *SE* = .06, *p* = .084 for both models), so we removed this link from the final models. The global judgment model showed a good fit to the data according to standard measures, χ^2^(4) = 9.60, *p* = .048, χ^2^/df = 2.40, SRMR = .05, RMSEA = .11 (even if the index should be lower than .05, it has been shown that it over-rejects models with small sample size; see [39]), CFI = .98, BIC = 62.26, AIC = 31.60, CAIC = 73.26. Fig 2A shows that global retrospective evaluation strongly predicted intentions (β = .85, *SE* = .05, *p* < .001), accounting for a large proportion of variance (*R*^*2*^ = .72). Conversely, the episodic judgment model (Fig 2B) showed unsatisfactory indices of fit, χ^2^(4) = 126.81, *p* < .001, χ^2^/df = 31.70, SRMR = .23, RMSEA = .56, CFI = .67, BIC = 179.47, AIC = 148.81, CAIC = 190.47. In addition, the fit of a model in which intentions were predicted by both retrospective measures (Fig 2C) was not better than the fit of the global judgment model, χ^2^_diff_ (1) = 3.43, *p* = .064, and the explained variance was the same (*R*^*2*^ = .72). Moreover, this model confirmed that the global retrospective evaluation is a much better predictor of intentions (β = .80, *SE* = .06, *p* < .001) than the episodic-derived evaluation (β = .10, *SE* = .05, *p* = .051).

Furthermore, the retrospective judgment model was the best-fitting model even when the episodic-derived evaluation score (computed with the first five recalled scenes) was replaced with a score computed with all the scenes or just using the most pleasant scene (see [36]; see S1 Appendix).

The good-fitting global judgment model also showed that expectancies influenced on-line evaluation (β = .61, *SE* = .11, *p* < .001), which, in turn, affected both kinds of retrospective evaluation measures (global retrospective evaluation: β = .61, *SE* = .06, *p* < .001; episodic-derived evaluation: β = .35, *SE* = .09, *p* < .001). Expectancies had also a significant direct effect on the global evaluation (β = .28, *SE* = .08, *p* < .001) and on the episodic-derived evaluation (β = .21, *SE* = .13, *p* = .031).

It is important to point out that we started from a general and independently-supported model frame, hypothesizing a sequential chain of relations between expectancies, online evaluation, memory, and intentions (e.g., [1, 2]) and that we teased out the memory component in order to clarify the specific contribution of episodic-derived vs. global evaluations via focused hypothesis testing. Given that some alternative models might be still viable within this general theoretical context, we tested the more plausible ones. In particular, alternative models including direct relations from expectancies to intentions or from online evaluation to intentions, showed non-significant paths (β = .10, *SE* = .08, *p* = .098, for the link between expectancies and intentions; β = .12, *SE* = .07, *p* = .107, for the link between online evaluation and intentions) and the fit was not improved (χ^2^_diff_ (1) = 2.71, *p* = .100; χ^2^_diff_ (1) = 2.57, *p* = .109, respectively).

These results support H1 and they show that the global judgment is the basis of intentions about similar future experiences, whereas the episodic-derived evaluation plays a small-to-null role, even if both are accessible. Moreover, the global retrospective evaluation was not related to the episodic-derived one, suggesting that these two potential memory bases of future intentions are independent.

## Study 2

Study 1 showed that when intentions refer to future hedonic experiences very similar to an overall past one, a global retrospective judgment about the past experience is the main memory source for the intention formation process. These findings supported H1: When the global judgment about the past experience was diagnostic for the prediction of the future experience, the global judgment was the preferred source for the intentions formation process, even if both global evaluation and episodic memories of the experience were available. Starting from this result, Study 2 tested H2: When the global retrospective judgment is not sufficiently diagnostic for future intentions then episodic-derived judgments should be also used as a basis in the intention formation process (provided that they are available).

In Study 2 we employed the same movie paradigm as in Study 1, but we asked participants to express intentions about experiences that were related only to specific parts of the past one: namely, future experiences involving only specific characters taken from the previously-seen movie. In this situation, only some parts of the movie are diagnostic for future intentions (i.e., the scenes in which the target character was actually present) and the global retrospective evaluation of the whole experience is only partially diagnostic, especially if the target character did not have a major role in the movie. Thus, in order to formulate sensitive future intentions, participants need to recall their episodic memories of the relevant parts of the movie.

In Study 2, intentions and episodic recollections referred to three characters of the movie. In order to test our specific hypotheses on the role of episodic contribution to future intentions, we focused on two minor characters with opposite evaluations (one positive and one negative). In addition, we included the main character of the movie (that was expected to be more related to global evaluation) to highlight the different role of episodic-derived vs. global retrospective judgments. The main character was Layale, who worked in a beauty salon in Beirut along with two other women and had a relationship with a married man. She was present very often in 28-minutes excerpt (46% of time) and was generally evaluated as attractive as the whole movie in a pre-test. The first minor character was Lili, an old woman with mental problems, quite distinctive and funny. Participants generally like her a lot according to the pre-test. The second minor character was Jamale, a regular customer of the beauty salon and a wannabe actress, worried about getting old. According to the pre-test, participants generally did not like her much. Both the minor characters appeared for a short time period in the excerpt (15% and 14% of the time). We expected that intentions involving a specific character would have been predicted not only by the global retrospective judgment, but also—and critically so—by episodic-derived evaluations of the target character. We did not expect the global evaluation of the movie to be completely inconsequential, given that the global evaluation may also depend on the evaluation of the scenes including the specific character, and given that the global judgment should be retrieved before the specific episodic memories according to the accessibility-diagnosticity model [13]. Additionally, we expected to replicate the finding showing that the global evaluation of the movie predicts future intentions about similar overall experiences.

### Method

#### Participants

One hundred and five undergraduates who had not seen the movie took part in the study (67% females, age: *M* = 22.82, *SD* = 5.12). Participants were recruited through announcements and they were randomly assigned to small group (ranging from 15 to 30 participants each) as in Study 1.

#### Procedure and materials

The first session of Study 2 was very similar to Study 1: Participants read the movie’s plot, they expressed their expectancies about the movie on the same 6-item questionnaire of Study 1 (Cronbach’s α = .89), and they watched the first twenty-eight minutes of the movie Caramel [28]. Given that in Study 1 we found no difference between the last online rating and the rating at the end of the movie, we assessed the online experience right after the end of the movie using the same 6-item expectancies questionnaire and just changing the verb tense (Cronbach’s α = .95). At the end of the first session, participants received written instruction not to talk about the study with other participants or friends and not to watch the rest of the movie nor collect information about it.

In the first session, participants were recruited and tested in small groups as in Study 1, whereas the second session was administrated online, through the Survey Monkey software (www.surveymonkey.com). A week after the first session, participants received an email containing the link to the online questionnaire, with the explicit request for an immediate compilation (all the participants completed the second session within the eighth day after the first session in 15–25 minutes). During this session, we collected the episodic measures separately for the three characters, the intentions related to experiences involving the three characters (i.e., specific future intentions), the global retrospective evaluation of the movie, and the intentions related to overall similar experiences (i.e., global future intention).

In the episodic measures, participants were presented with the pictures of the three characters (the main character, the minor character 1, and the minor character 2), one by one, and in a random order. They were asked to report as many scenes as possible in which the specific character was present (up to five), and to evaluate each of them for pleasantness and vividness on 7-point scales (from not at all to very much). Specific intentions for each character were assessed using a 5-item questionnaire (main character: Cronbach’s α = .89; minor character 1: Cronbach’s α = .92; minor character 2: Cronbach’s α = .91). These items were designed adapting the global intentions items to the specific character. In particular, participants were asked to report on a 7-point scale (ranging from not at all to very much) how much they were interested in discovering how the story of the character continued, in watching a movie focused on that character, in watching a movie with the same actress as main character, and if they were willing to suggest to a friend a movie focused on that character, and a movie with the same actress as main character. Global retrospective evaluation (Cronbach’s α = .96) and global future intentions (Cronbach’s α = .92) were assessed using the same questionnaires of Study 1. The order of the memory-related measures was counterbalanced, with the specific future intentions measure interleaved and the global future intentions collected always at the end. At the end of the session, participants were asked to evaluate the three characters along three items (how much they liked the character, how nice and how funny the character was, ranging from not at all to very much) as manipulation check. All the measures’ scores (expectancies, on-line evaluation, retrospective evaluation, specific and global future intentions) were obtained by averaging responses to the items of each measure.

### Results and discussion

Seven participants confused a character with another, and seven failed to report scenes involving the minor character 2, thus the subsequent analyses were based on the sample of participants who correctly recollected at least one scene for each character (*n* = 91).

Participants recalled on average 3.91 scenes out of five in which the main character was present (*SD* = 1.28), 3.45 scenes in which the minor character 1 was present (*SD* = 1.16), and 1.97 scenes in which the minor character 2 was present (*SD* = 1.11). The accuracy of the retrieved scenes was high, ranging from 95% for the scenes involving the main character, to 90% for the scenes involving the minor character 2 (see Study 1 for the scoring criteria). As in Study 1, scenes were evaluated by two independent judges with good inter-rater agreement (Cohen’s kappa = .74, computed on a random selection—10%—of retrieved scenes). Memories involving the main character and the minor character 1 were evaluated as quite vivid, significantly above the mid-point of the scale (*M*_vividness_ = 4.44, *SD* = 1.39), *t*(90) = 3.06, *p* = .003, and (*M*_vividness_ = 4.60, *SD* = 1.35), *t*(90) = 4.26, *p* < .001, respectively, whereas recollections involving the minor character 2 were evaluated below the mid-point of the scale (*M*_vividness_ = 3.71, *SD* = 1.40), *t*(90) = 1.97, *p* = .052. These results show that episodic recollections were highly accessible for the main character and the minor character 1, but the episodic recollection involving the minor character 2 was more difficult (we will discuss this further in the paper). An episodic-derived evaluation score was computed for each character, by averaging the pleasantness ratings of the scenes recalled by participants.

The order of administration of memory measures did not affect episodic-derived evaluations, main character, *t*(89) = 0.43, *p* = .668, minor character 1, *t*(89) = 0.54, *p* = .589, minor character 2, *t*(89) = 0.73, *p* = .470; global retrospective evaluations, *t*(89) = 1.78, *p* = .078; specific future intentions, main character, *t*(89) = 0.56, *p* = .574, minor character 1, *t*(89) = 0.12, *p* = .908, minor character 2, *t*(89) = 0.61, *p* = .546; and global future intentions, *t*(89) = 1.67, *p* = .098, therefore it was not considered any further.

As in Study 1, path analysis was used for hypothesis testing. The matrix of correlations between variables is reported in the S1 Appendix. The models were specified by following Study 1, including expectancies, online evaluation, episodic-derived evaluations for each character, global retrospective evaluation, specific future intentions for each character, and global future intentions. In all the models, we tested the influence of expectancies on the online evaluation, and the influence of expectancies and on-line evaluation on all the memory measures (after that, we removed the non-significant links and re-estimated the models). The three episodic-derived measures and the three specific intentions measures were supposed to be correlated in all the models: Given that characters rarely appeared alone in the movie, we expected the evaluations to be related. In line with the results of Study 1, we supposed that global retrospective evaluation was able to predict global future intentions. We included in the models also a possible relation between specific intentions about the main character and global future intentions, given that the movie is mainly based on the main character and the main character appeared often during the movie.

**Fig 3:**
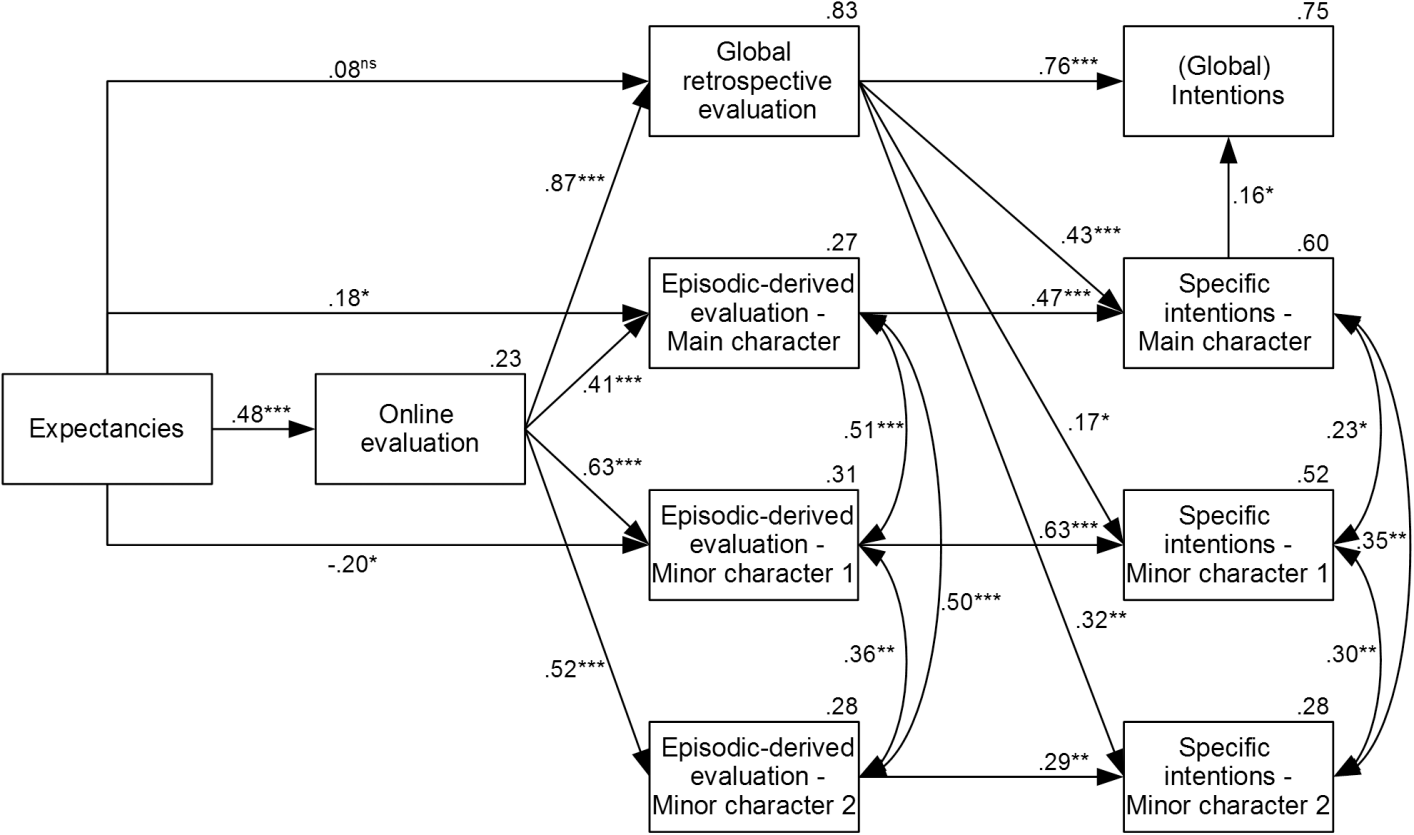
Path analysis model in Study 2. Path analysis model of Study 2, in which both global judgment and episodic-derived evaluations of each character predicted specific intentions. Numbers close to the arrows are standardized path coefficients. Those above the boxes indicate explained variance (*R*^*2*^). Significance levels are as follows: * *p* < .05, ** *p* < .01, *** *p* < .001.

The first model tested the hypothesis (H2) that both the episodic-derived evaluations and the global retrospective evaluation predicted specific intentions (see Fig 3). That is, the global retrospective evaluation was supposed to predict both global and specific intentions, while the episodic-derived evaluations of each character were supposed to predict the specific intentions related to the corresponding character (Fig 3). The model showed a good fit to the data according to the standard measures, χ^2^(23) = 31.88, *p* = .103, χ^2^/df = 1.39, SRMR = .05, RMSEA = .07, CFI = .99, BIC = 176.22, AIC = 95.88, CAIC = 208.22. Results showed that main character intentions were predicted, in a similar way, by global retrospective evaluation (β = .43, *SE* = .07, *p* < .001) and by episodic-derived evaluation (β = .47, *SE* = .07, *p* < .001), accounting for the 60% of the variance. The minor character 1 intentions were predicted especially by episodic-derived evaluation (β = .63, *SE* = .07, *p* < .001) and, to a lower extent, by global retrospective evaluation (β = .17, *SE* = .09, *p* = .038), accounting for the 52% of the variance. The minor character 2 intentions were predicted in a similar way by both global retrospective evaluation (β = .32, *SE* = .08, *p* = .002) and episodic-derived evaluation (β = .29, *SE* = .08, *p* = .002), accounting for the 28% of the variance. In line with Study 1, global future intentions were strongly predicted by global retrospective evaluation (β = .76, *SE* = .07, *p* < .001), and slightly by the main character specific intentions (β = .16, *SE* = .07, *p* = .020), accounting for a great amount of variance (75%). In addition, expectancies influenced online evaluation (β = .48, *SE* = .12, *p* < .001), which, in turn, influenced all memory measures (βs > .41, *p*s < .001). Direct effects of expectancies on retrospective measures were found for episodic-derived evaluation of the main character (β = .18, *SE* = .11, *p* = .049) and of the minor character 1 (β = -.20, *SE* = .14, *p* = .031), while for the global retrospective evaluation the effect was weaker than in Study 1 and nonsignificant (β = .08, *SE* = .06, *p* = .112). In summary, these findings show that, when future intentions refer to a specific part of the past experience, both episodic-derived evaluations and global retrospective evaluation contribute to the intentions formation process, in agreement with H2.

In order to better understand whether the episodic-derived evaluation or the global retrospective evaluation suffice to predict a specific intention, we tested two alternative models. In the global judgment model, specific intentions were supposed to be predicted only by the retrospective global evaluation, whereas in the episodic model specific intentions were supposed to be predicted only by the episodic-derived evaluation of the corresponding character. The remaining parts of the models were unchanged. Results showed unacceptable fit indices according to the standard measures, both for the global judgment model, χ^2^(26) = 107.17, *p* < .001, χ^2^/df = 4.12, SRMR = .13, RMSEA = .19, CFI = .88, BIC = 237.98, AIC = 165.17, CAIC = 266.98, and for the episodic-derived model, χ^2^(26) = 61.66, *p* < .001, χ^2^/df = 2.37, SRMR = .12, RMSEA = .12, CFI = .95, BIC = 192.47, AIC = 119.66, CAIC = 221.47. Moreover, the initial model we tested (the one including the influence of both kinds of predictors; Fig 3) showed a better fit to the data than either the global judgment model, χ^2^_diff_ (3) = 75.29, *p* < .001, or the episodic-derived model, χ^2^_diff_ (3) = 29.78, *p* < .001.

As in Study 1, the models we tested are theoretically-grounded and based on the derived hypotheses. As noted before for Study 1, other alternative models are still possible, although they are more complex and they do not necessarily improve the model fit. Indeed, models in which global and specific intentions were predicted by expectancies or by online evaluation did not improve the fit with respect to our final model (χ^2^_diff_ (4) = 2.74, *p* = .603; χ^2^_diff_ (4) = 6.1521, *p* = .188, respectively). Moreover, a model in which episodic-derived evaluation of the main character (that should be more consistent with the whole experience) predicted global intentions showed a nonsignificant path (β = .04, *SE* = .07, *p* = .553) and did not improve the fit of the model (χ^2^_diff_ (1) = 0.32, *p* = .572).

Taken together, these results show that the episodic-derived evaluations are needed to predict specific intentions and that the global retrospective evaluation of the movie does not suffice. Therefore, Study 2 supported our second hypothesis: When the global judgment was not sufficiently diagnostic for future intentions, relevant episodic traces were recollected and used as a source for the judgment, along with the global evaluation. In addition, we replicated the results of Study 1, by showing that the global judgment was the more important source of information for similar future experiences.

The extent to which global and episodic-derived evaluations affected specific intentions varied across the three characters considered: The episodic-derived evaluation was the preferred source for the intentions related to the minor character 1 (β = .63 vs. β = .17), while both sources affected in a similar way the intentions related to the main character (β = .47 vs. β = .43, respectively) and to the minor character 2 (β = .29 vs. β = .32, respectively). Although we did not formulate *a priori* hypotheses about specific characters, the accessibility-diagnosticity framework can offer a reasonable post-hoc account of the influence of the two memory sources varying across characters. In the case of the minor character 1, relevant episodic memories were both accessible and diagnostic, so participants simply relied mainly on them to express intentions. Coming to the minor character 2, as Lynch pointed out [12], information low in accessibility can be considered also less diagnostic and it may be distrusted in the judgment. Therefore, the low accessibility of information about the minor character 2 could have led participants to demote this information as less diagnostic (1.97 scenes recalled on average vs. 3.45 scenes related to the minor character 1). As a consequence, participants relied also on global retrospective evaluations to formulate specific intentions about this specific character. As for the main character, the retrospective global judgment of the movie was at least partially diagnostic for future intentions, given the prominent role of the main character in the movie. At the same time, relevant episodic memories were highly accessible (participants were able to recollect 3.91 related scenes on average) and also diagnostic. As a consequence, participants relied on both these memory sources.

Regardless the character-related variation, however, the fundamental finding of Study 2 shows that when future intentions were character-specific, episodic-derived evaluations were always taken into account and had a critical role in the prediction of future intentions, while episodic-derived evaluations had no role when the future intentions referred to an overall experience similar to the past one.

## General discussion

In two empirical studies, we investigated the specific memory bases of future intentions about a hedonic experience. We found that future intentions were always based on memory of similar past experiences, but the specific memory sources of future intentions (global judgments vs. episodic memories) depended on the relative diagnosticity of these sources. When future intentions referred to hedonic events closely related to an overall past experience (e.g., watching a movie sequel), individuals relied only on global retrospective evaluations of the past experience despite the accessibility of both the global judgment and the episodic memories about the past experience (Study 1). However, when the global retrospective evaluation was not sufficiently diagnostic for the future intentions, because the intentions referred to events related only to specific parts of the past experience (watching a movie centred on a minor character of the previously seen movie), specific episodic memories contributed in a critical way to the expression of future intentions (Study 2).

In addition, in line with the literature, we observed the indirect effect of expectation on retrospective evaluation through online evaluations (see [1]) and the indirect effect of online evaluation on future intentions. This latter evidence is fully consistent with previous work (e.g., [1, 40]) and provides further support for the hypothesis that future intentions are based on memory of past experiences and only indirectly on actual experiences.

However, these findings go beyond the observation that future intentions do depend on memory of the previous experience (and not on how the past experience was perceived), and they show, for the first time, that two distinct memory sources contribute differentially to diverse kinds of future intentions, thus shedding light on the memory sources underlying future intentions. More generally, the results show that the accessibility-diagnosticy framework [3] is generally tenable also in the field of future intentions about hedonic experiences and provide novel support for its predictions.

Importantly, our findings also support the view that global judgments and episodic-derived evaluations represent two distinct memory sources for future intentions, in line with Hastie and Park’s distinction [4]. Indeed, in Study 1, global judgments were not related with episodic-derived evaluations, once controlling for the influence of expectancies and online evaluations. Additionally, even if we did not directly demonstrate that global judgments were formed on-line during the hedonic experience, they were stored in memory, and accessed when they were required to form intentions about the future, our findings are supportive of this view, showing that online experience is strongly associated with the global retrospective judgment, with online evaluations strongly predicting global evaluations both in Study 1 and in Study 2.

From a more general point of view, our findings suggest that detailed episodic simulations of future experiences are not necessary to express intentions when there is a good match between the past and the future experiences. If this is the case, individuals may rely only on global judgments in the intention formation process even when relevant episodic memories are still available. In line with these findings, scholars who extensively studied real-world decision making (e.g., [41]) postulate that effortful and time-consuming mental simulations of past or future scenarios are infrequent and are typically carried out in anomalous or uncertain situations, in which recognition-based decision-making routines cannot be straightforwardly applied. This seems to contrast with a shared view in episodic future thinking research, which postulates that decisions about the future are based on rather detailed simulations of future scenarios based on episodic building blocks, more or less shaped by semantic knowledge ([42–44]; for a review, [45]). Our findings show that intention formation may not always require simulating the future ‘episodically’ and point to the need of carrying out a deeper appraisal of the extent to which individuals actually build detailed simulations of future scenarios to guide their future decision behavior (see also [45–46]). Interestingly, some recent neuropsychological investigations show that amnesic participants can make decisions similar to normal controls about the future even if their episodic memory is impaired, systematically discounting future rewards despite being unable to construct the details of either past or future events [47].

Although our studies have begun to shed light on the memory underpinnings of future intentions, more research is still needed to get a complete picture. First, given that the entertainment experience could be different according to the kind of movies and the audience (e.g., [48]), investigating different kinds of movies (i.e., belonging to different genres) and different hedonic experiences would be useful and informative. It is worth noting, however, that we replicated the sequential chain between expectancies, online evaluation, memory, and intentions that has been observed with completely different hedonic experiences, such as a vacation [1] and the fruition of public transportation [2]. This suggests that the pattern of relations between memory and intentions might be generalized to other hedonic experiences, but actual replication of our results with further different experiences is still needed.

Second, even if the link between intentions and actual behavior were not the topic under scrutiny in our studies, a natural extension of our research would be to appraise the consistency between intentions and actual behavior.

Third, although our findings are based on an established paradigm to investigate future intentions, the results are correlational and caution needs to be exerted in their interpretation. For instance, the relationship between memory and intentions might be also reciprocal. Nevertheless, there is independent evidence supporting the relation from memory to intentions. Global (retrospective) evaluations seem to precede intentions, which is a necessary condition for causality: On-line judgments leading to global retrospective evaluations can be generated even without having any intention about the future (e.g., [4]) and summary evaluations can be generated even automatically from experience and eventually used in the future (e.g.,[5]). Moreover, other studies showed that participants were more willing to repeat the experience that they remember to be less painful and not the one that the online measures suggested to be less painful (e.g., [40]), suggesting that memory, and not the actual experience, affects intentions. As already reported, there are other studies showing that memory mediates the effect of online evaluation of the experience on future intentions (e.g., [1,2]). Finally, in the present work we also tested alternative models including direct relations from expectancies to intentions or from online evaluation to intentions and these models showed nonsignificant paths and worse fit indices. Nevertheless, we point out the need of further investigating the memory bases of future intentions with experimental methods in order to bring additional support to our interpretation of the findings.

Finally, there are some minor methodological limitations that need to be addressed in future research. First, the sample composition was gender-unbalanced. Although gender was not expected to have any role in relation to our hypotheses given that the specific intention-formation and memory mechanism under analysis are assumed to be relatively stable and general, a sample with an equal gender distribution would be preferable. Second, given the time lag between the two sessions, it cannot be excluded that participants gathered information about the movie and, even if we kindly asked participants not to do so, we cannot really control what participants did outside the laboratory. Future research should address this issue using hedonic experiences for which it is not possible to obtain external information and use a sample of participants who do not know each other in order to avoid any risk of diffusion.

In conclusion, the present research provided a novel theoretical and empirical contribution on the memory bases of future intentions by bridging work on memory-based judgments and future intentions. Beyond offering novel empirical evidence on the memory bases of future intentions and providing related theoretical insights, our findings have broader implications for researchers in memory and judgment and decision making. Thus, recalling the title of our paper, we hope that there will be sequels to our studies, which will provide further knowledge on a research topic that has broad theoretical and applied relevance.

## Supporting information

S1 Appendix Additional analyses. The appendix reported alternative models for Study 1 and correlation matrices for Study 1 and 2.
(DOCX)

S1 Dataset Study 1 dataset. (XLSX)

S2 Dataset Study 2 dataset. (XLSX)
