# Supplementary material for: Memory underpinnings of future intentions: Would you like to see the sequel?
Source: PLoS One. 2017 Apr 27;12(4):e0176624. doi: 10.1371/journal.pone.0176624 (PMC5407789; doi:10.1371/journal.pone.0176624)
Supplement: S1 Appendix — The appendix reported alternative models for Study 1 and correlation matrices for Study 1 and 2. (DOCX) [file pone.0176624.s001.docx]

S1 Appendix

Memory Underpinnings of Future Intentions: Would You Like to See the Sequel?

Table of contents

1. Study 1 supplementary analysis
2. Table of correlations of Study 1 and 2

**1. Study 1 supplementary analysis**

*Sensitivity analysis.* We estimated the same models presented in Study 1, replacing the episodic-derived index (mean of evaluation of the first five movie scenes recalled) with indices derived by using the evaluations of a different number of scenes: all the scenes recollected by each participant, only the first scene, only the first two scenes, only the first three scenes, only the first four scenes, only the most pleasant scene. Estimation results are presented in Table 1. The findings confirmed the results presented in the paper.

**Table 1. Study 1 models (global, episodic, and full path) using different indices as the measure of episodic-derived evaluation.**

|  |  | Fit indices | | | | | | | Intentions predictor | | | *R^2^* |
| --- | --- | --- | --- | --- | --- | --- | --- | --- | --- | --- | --- | --- |
|  |  | **χ^2^, *p*** | **χ^2^*/df*** | ***SRMR*** | ***RMSEA*** | ***AIC*** | ***CFI*** | ***BIC*** | ***β*** | ***SE*** | ***p*** |  |
| All the scenes | |  |  |  |  |  |  |  |  |  |  |  |
|  | Global model | 11.52, p = .021 | 2.88 | .05 | .13 | 33.52 | .98 | 64.18 | .85 | .05 | <.001 | .72 |
|  | Episodic model | 127.07, p<.001 | 31.77 | .23 | .51 | 149.07 | .67 | 179.73 | .50 | .08 | <.001 | .25 |
|  | Full path model | 9.55, p = .023 | 3.18 | .04 | .14 | 33.55 | .98 | 67.00 | g: .81 | .06 | <.001 | .72 |
|  |  |  |  |  |  |  |  |  | e: .08 | .05 | .131 |  |
| First scene | |  |  | . |  |  |  |  |  |  |  |  |
|  | Global model | 8.46, p = .076 | 2.11 | .04 | .10 | 30.46 | .99 | 61.12 | .85 | .05 | < 001 | .72 |
|  | Episodic model | 142.83, p<.001 | 35.71 | .28 | .54 | 164.83 | .60 | 195.50 | .36 | .05 | <.001 | .13 |
|  | Full path model | 2.75, p = .431 | 0.92 | .02 | .00 | 26.75 | 1.00 | 60.20 | g: .81 | .05 | <.001 | .73 |
|  |  |  |  |  |  |  |  |  | e: .12 | .03 | .016 |  |
| First two scenes | |  |  |  |  |  |  |  |  |  |  |  |
|  | Global model | 6.65, p = .156 | 1.66 | .03 | .07 | 28.65 | .99 | 59.31 | .85 | .05 | <.001 | .72 |
|  | Episodic model | 137.44, p<.001 | 34.36 | .26 | .53 | 159.44 | .63 | 190.10 | .39 | .06 | <.001 | .15 |
|  | Full path model | 2.65, p = .450 | 0.88 | .02 | .00 | 26.65 | 1.00 | 60.10 | g: .81 | .05 | <.001 | .73 |
|  |  |  |  |  |  |  |  |  | e: .10 | .04 | .045 |  |
| First three scenes | |  |  |  |  |  |  |  |  |  |  |  |
|  | Global model | 6.55, p = .162 | 1.64 | .03 | .07 | 28.55 | .99 | 59.21 | .85 | .05 | <.001 | .72 |
|  | Episodic model | 130.29, p<.001 | 32.57 | .24 | .52 | 152.29 | .65 | 182.95 | .45 | .07 | <.001 | .20 |
|  | Full path model | 3.07, p = .380 | 1.02 | .02 | .01 | 27.07 | >.99 | 60.52 | g: .81 | .06 | <.001 | .72 |
|  |  |  |  |  |  |  |  |  | e: .10 | .05 | .057 |  |
| First four scenes | |  |  |  |  |  |  |  |  |  |  |  |
|  | Global model | 7.86, p = .097 | 1.96 | .04 | .09 | 29.86 | .99 | 60.52 | .85 | .05 | <.001 | .72 |
|  | Episodic model | 128.85, p<.001 | 32.21 | .24 | .51 | 150.85 | .66 | 181.51 | .47 | .08 | <.001 | .22 |
|  | Full path model | 4.07, p = .254 | 1.36 | .02 | .05 | 28.07 | >.99 | 61.52 | g: .80 | .06 | <.001 | .72 |
|  |  |  |  |  |  |  |  |  | e: .11 | .05 | .044 |  |
| Most pleasant scene | |  |  |  |  |  |  |  |  |  |  |  |
|  | Global model | 19.86, p<.001 | 4.97 | .07 | .18 | 41.86 | .96 | 72.53 | .85 | .05 | <.001 | .72 |
|  | Episodic model | 129.31, p<.001 | 32.33 | .22 | .51 | 151.31 | .67 | 181.97 | .54 | .08 | <.001 | .29 |
|  | Full path model | 19.21, p<.001 | 6.40 | .06 | .21 | 43.21 | .96 | 76.66 | g: .82 | .06 | <.001 | .72 |
|  |  |  |  |  |  |  |  |  | e: .05 | .06 | .359 |  |

Indices of episodic-derived evaluations were based on all the recollections, the first recollection, the first two recollections, the first three recollections, the first four recollections, and the most pleasant recollection. Fit indices, standardized coefficient of predictors along with *SE* and *p*, and *R^2^* are reported. Predictors of intentions were global evaluations in the global models, episodic-derived evaluations in the episodic models, both global and episodic-derived evaluations in the full path model (the abbreviation “g” refers to the coefficients of the global evaluation predictor, and “e” refers to the coefficients of the episodic-derived predictor).

**2. Correlation Tables**

Pearson’s pairwise correlations between all the measures used in path analyses are presented in Table 2 (for Study 1) and in Table 3 (for Study 2).

**Table 2. Pearson's Pairwise Correlations for all the Models Estimated in Study 1.**

| Measure | 1 | 2 | 3 | 4 | 5 |
| --- | --- | --- | --- | --- | --- |
| 1. Expectancies | ̶ |  |  |  |  |
| 1. On-line evaluation | .61 | ̶ |  |  |  |
| 1. Episodic-derived evaluation | .43 | .48 | ̶ |  |  |
| 1. Global retrospective evaluation | .65 | .78 | .49 | ̶ |  |
| 1. Intentions | .61 | .71 | .49 | .85 | ̶ |

All correlations were significant at the .001 level (two-tailed).

**Table 3. Pearson's Pairwise Correlations for all the Models Estimated in Study 2.**

| Measure | 1 | 2 | 3 | 4 | 5 | 6 | 7 | 8 | 9 | 10 |
| --- | --- | --- | --- | --- | --- | --- | --- | --- | --- | --- |
| 1. Expectancies | ̶ |  |  |  |  |  |  |  |  |  |
| 1. On-line evaluation | .48^*^ | ̶ |  |  |  |  |  |  |  |  |
| 1. Episodic-deriv. eval. – main char. | .41^*^ | .49^*^ | ̶ |  |  |  |  |  |  |  |
| 1. Episodic-deriv. eval. – minor char. 1 | .13 | .53^*^ | .59^*^ | ̶ |  |  |  |  |  |  |
| 1. Episodic-deriv. eval. – minor char. 2 | .33^*^ | .52^*^ | .63^*^ | .52^*^ | ̶ |  |  |  |  |  |
| 1. Global retrospective evaluation | .50^*^ | .91^*^ | .54^*^ | .53^*^ | .55^*^ | ̶ |  |  |  |  |
| 1. Specific intentions – main char. | .34^*^ | .58^*^ | .72^*^ | .51^*^ | .59^*^ | .67^*^ | ̶ |  |  |  |
| 1. Specific intentions – minor char. 1 | .12 | .45^*^ | .44^*^ | .72^*^ | .49^*^ | .49^*^ | .51^*^ | ̶ |  |  |
| 1. Specific intentions – minor char. 2 | .13 | .36^*^ | .47^*^ | .41^*^ | .51^*^ | .47^*^ | .61^*^ | .52^*^ | ̶ |  |
| 1. (Global) Intentions | .45^*^ | .80^*^ | .54^*^ | .57^*^ | .51^*^ | .86^*^ | .67^*^ | .53^*^ | .49^*^ | ̶ |

* correlations were significant at the .01 level (two-tailed)
